# Genome-Wide Analysis of the SAUR Gene Family and Its Expression Profiles in Response to Salt Stress in *Santalum album*

**DOI:** 10.3390/plants13101286

**Published:** 2024-05-07

**Authors:** Qing Zhu, Haoyue Zheng, Xu Hu, Yi Liu, Xinyi Zheng, Libei Li, Minqiang Tang

**Affiliations:** 1 Key Laboratory of Genetics and Germplasm Innovation of Tropical Special Forest Trees and Ornamental Plants (Ministry of Education), Hainan Key Laboratory for Biology of Tropical Ornamental Plant Germplasm, Collaborative Innovation Center of Ecological Civilization, School of Tropical Agriculture and Forestry, Hainan University, Haikou 570228, China; 2 College of Advanced Agriculture Sciences, Zhejiang A&F University, Hangzhou 311300, China

**Keywords:** SAUR gene family, *Santalum album*, transcriptome analysis, salt stress, RT-qPCR

## Abstract

The SAUR (small auxin-up RNA) family constitutes a category of genes that promptly respond to the hormone auxin and play a pivotal role in diverse biological processes encompassing plant growth and the response to abiotic stress. *Santalum album* L., a semi-parasitic evergreen tree, is renowned for its economically valuable essential oils, positioning it among the most prized tree species. In this study, a meticulous identification and comprehensive analysis of 43 *SAUR* genes was conducted within *S. album*. Based on phylogenetic relationships, the *SaSAUR* genes were systematically categorized into five groups. A collinearity analysis revealed intriguing insights, disclosing 14 segmental duplications and 9 tandem duplications within the *SaSAUR* genes, emphasizing the pivotal role of duplication in the expansion of this gene family. Noteworthy variations in the expression levels of *SaSAUR* genes were observed by delving into the *SaSAUR* transcriptome data from various tissues, including leaves, roots, and heartwood, as well as under salt-stress conditions. Notably, *SaSAUR08* and *SaSAUR13* were significantly upregulated in heartwood compared with roots and leaves, while *SaSAUR18* was markedly more expressed in roots compared with heartwood and leaves. Furthermore, *SaSAUR27* and *SaSAUR28* were found to respond closely to salt stress, hinting at their potential involvement in the salt-stress response mechanism. This research offers a comprehensive investigation of *SAUR* genes in *S. album* and establishes a foundation for future exploration of the *SAUR* gene family, particularly its relation to growth and salt-stress responses.

## 1. Introduction

*Santalum album* L. is an evergreen semi-parasitic tree belonging to the Santalum genus in the Santalaceae family and is mainly distributed in Southeast Asia and the Pacific region [1]. As *S. album* is one of the tree species with remarkable economic worth, all its parts can be utilized as raw materials. The heartwood oil is renowned for its unique aroma [2] and has vital roles as an anti-cancer [3], anti-inflammatory [4], and antioxidative substance [5]. Therefore, it has widespread application in various industries, including in perfumery, pharmaceuticals, cosmetics, etc. Heartwood oil is often referred to as “liquid gold” [6]. *S. album*’s heartwood possesses a remarkable hardness and a beautiful texture, making it one of the important raw materials for wood carving and the production of expensive furniture [7]. Therefore, *S. album* currently stands as one of the most widely commercially developed and utilized tropical tree species. In recent years, the demand for its heartwood and sandalwood essential oils has been increasing in both domestic and foreign markets; however, the growth rate and heartwood formation of *S. album* is relatively slow. Consequently, the prices of *S. album* have risen dramatically, leading to the overexploitation of this resource [8]. Therefore, proper cultivation and protection management measures of the sandalwood tree is of the utmost need at this time [9].

SAUR (small auxin-up RNA) belongs to one of the three major early auxin-response genes [10]. Early auxin-response genes can be rapidly activated and transcribed upon auxin induction without the need for new protein synthesis. Moreover, the molecular weights of the proteins encoded by *SAUR* genes are generally small, ranging from 9–30 kDa [11]. Auxin, as the earliest discovered plant hormone, can promote rooting, regulate shoot development, and control cell division and differentiation [12]. It plays a crucial role in plant growth and stress resistance [13]. The *SAUR* genes were first discovered in soybean hypocotyls that were stimulated to elongate by auxin [14]. Subsequently, *SAURs* have been found in a variety of plants, including *Arabidopsis* (*Arabidopsis thaliana*) [15], rice (*Oryza sativa*) [16], sorghum (*Sorghum bicolor*) [17], and pineapple (*Ananas comosus*) [18]. Among the early auxin-responsive genes, the *SAUR* gene family has the largest number of genes and a high degree of structural similarity [19]. Most *SAURs* contain only one exon and no introns. They are found in clusters on the chromosome, indicating that high frequencies of tandem and segmental duplications have occurred during evolution [20]. Auxin regulates gene expression by the binding of auxin response factor (ARF) to auxin response elements (AuxREs) in the promoter region of auxin-responsive genes [21]. The number and diversity of AuxREs in different *SAURs* vary significantly. Nine AuxREs have been identified in rice [16] and seven in tomato [22]. The mRNA encoded by the *SAURs* is highly unstable due to the presence of a conserved downstream element (DST) in its 3′ untranslated region (3′ UTR) [23]. This instability reduces the association between auxin and their regulated *SAUR* genes.

The *SAUR* gene family is involved in multiple processes in plants, i.e., plant growth and development. It plays an important role in responding to changes in auxins [24], affecting their synthesis and transport, as well as cell elongation and senescence resistance [25]. In *Arabidopsis thaliana*, overexpression of the *AtSAUR36* [26] and *AtSAUR41* [27] genes results in the significant elongation of hypocotyl epidermal cells, while the *AtSAUR36* gene responds to leaf senescence [28]. The *SAUR* gene family is involved in abiotic stress tolerance [29], and many *SAUR* genes are down-regulated under abiotic stress as an adaptation to adverse environments [30]. In the model species *Arabidopsis thaliana*, seedlings from *AtSAUR41* overexpression lines showed higher sensitivity to salt stress, while the *AtSAUR41* subfamily of genes was found to be associated with salt tolerance [31]. In the perennial plant *Medicago sativa*, several *SAUR* genes have been associated with abiotic-stress tolerance. Specifically, *MsSAUR14*, *MsSAUR94*, *MsSAUR297*, *MsSAUR306*, and *MsSAUR254* respond to both drought and salinity stress [32]. In *Glycine max*, it was discovered that *GmSAUR299* may be related to drought tolerance [33]. In poplar, the PagWOX11/12a proteins directly bind to the promoter of the *SAUR36* gene, while enhanced binding is observed under salt stress, thereby improving the salt tolerance [34]. Expression of the poplar genes *PtSAUR12*, *PtSAUR34*, *PtSAUR54*, *PtSAUR67*, *PtSAUR91*, and *PtSAUR97* was up-regulated at low temperatures [35]. These findings reflect the significant role of the *SAUR* gene family in different plants and trees; however, a comprehensive genome-wide identification of the *SAUR* gene family in *S. album* has yet to be documented.

In this study, bioinformatics methods were employed to identify the members of the *SAUR* gene family in *S. album*, while also delving into their molecular characteristics, chromosomal distribution patterns, gene structures, conserved motifs, and their evolutionary and functional intricacies. Leveraging transcriptome data, we further investigated the expression profiles of *SAUR* genes across various tissues, including heartwood, root, and leaf, as well as their response to salt stress. In conclusion, this study provides valuable resource information for understanding the regulatory roles of *SAUR* gene family members in *S. album*’s growth and stress tolerance.

## 2. Materials and Methods

### 2.1. Identification of SAUR Genes and Chromosomal Distribution

A hidden Markov model profile of the SAUR DNA-binding domain (PF00642) was downloaded from the Pfam database (http://pfam.xfam.org/) [36]. The whole genome sequence and annotation files of *S. album* employed in this study were downloaded from the National Genomics Data Center of China under accession number GWHCBHT00000000, which is publicly accessible at https://ngdc.cncb.ac.cn/gwh/Assembly/37800/show (accessed on 9 December 2023). The initial members of the *SAUR* gene family were identified in the genome sequence of *S. album* through an HMM search (E < 1 × 10^−5^) [37]. Subsequently, we utilized the SMART (http://smart.embl.de/smart/batch.pl/, accessed on 11 December 2023) [38] and CDD online websites (https://www.ncbi.nlm.nih.gov/cdd/, accessed on 11 December 2023) [39] to meticulously screen the initial family members for any potential redundancy. The distribution of SAUR proteins on chromosomes was mapped using the MapChart version 5.4.6 software based on the data from the *S. album* genome annotation files [40]. The *SAUR* genes of *S. album* were renamed based on their chromosomal location, following the nomenclature of the *Arabidopsis thaliana SAUR* gene family members [41].

### 2.2. Phylogenetic Analysis and Classification of SAUR Genes

Firstly, the protein sequences of the *SAUR* gene family members of *Arabidopsis thaliana* were downloaded from the TAIR database website (http://www.arabidopsis.org/, accessed on 1 December 2023). Subsequently, multiple sequence alignment was performed on SAUR proteins from *A. thaliana* and *S. album* using the MAFFT version 7 software [42]. The resulting alignment was then used to construct a phylogenetic tree using the IQ-TREE version 2 software and the maximum likelihood (ML) method [43]. The phylogenetic tree was bootstrapped 1000 times with default parameters, resulting in the final phylogenetic tree file. The phylogenetic tree was uploaded to the iTOL website (https://itol.embl.de) for visualization and information annotation [44]. The *SAUR* gene family members of *S. album* were classified based on the branch clustering of the phylogenetic tree.

### 2.3. Gene Duplication and Analysis of Protein Properties 

The Multiple Collinearity Scan toolkit X version 1 (MCScanX) [45] with default parameters was used to identify gene duplications, which were then visualized by using the One Step MCScanX tool in TBtools [46]. Non-synonymous substitutions (Ka) and synonymous substitutions (Ks) were determined for each duplicated *SAUR* gene pair using the KaKs_Calculator 2.0 [47]. The length of amino acids, theoretical isoelectric point (pI), and molecular weight of the SAUR proteins were analyzed using the online tool ExPASy (https://web.expasy.org/protparam/, accessed on 13 December 2023) [48]. The subcellular localization of the SAUR proteins was predicted using the Plant-mPLoc predictor (http://www.csbio.sjtu.edu.cn/bioinf/plant-multi/, accessed on 13 December 2023) [49]. Conserved motifs in the *S. album* SAUR proteins were identified using the online software Multiple Em for Motif Elicitation (MEME) suite 5.5.5 (https://meme-suite.org/meme/tools/streme, accessed on 13 December 2023), with a maximum of 10 motifs [50]. Additionally, we extracted exon and intron positions from the *S. album* genome annotation file. We used the TBtools Gene Structural View (Advanced) [46] to visualize the identified conserved motifs and gene structures.

### 2.4. Transcriptome Data and Expression of SAUR Genes

To analyze the gene functions in growth and development, we downloaded the transcriptome data for various *S. album* tissues (PRJNA243306, PRJNA360335, PRJNA192417) from the NCBI (National Center for Biotechnology Information, http://www.ncbi.nlm.nih.gov/, accessed on 1 December 2023). To investigate the mechanism of the salt-stress response in *S. album* mediated by the *SAUR* genes, approximately 50 cm tall sandalwood seedlings were treated with 50 mmol/L and 100 mmol/L NaCl solutions, while the blank control group was treated with an aqueous solution. The third or fourth fully stretched leaf samples were taken at 0, 12, 24, and 48 h intervals. All seedlings were placed under controlled growth conditions in an artificial climate chamber set at a constant temperature of 26 °C and 50% relative humidity, with 16 h of light exposure (200 μmol photons m^−2^ s^−1^, 26 °C) and 8 h in the dark. The experiment was repeated three times, resulting in a total of 36 samples. All the samples were collected, directly frozen in liquid nitrogen, and stored at −80 °C for RNA extraction. Total RNA was extracted from the leaves of each treatment using the TRIzol reagent. The RNA quality and quantity were checked using an Agilent 2100 Bioanalyzer, and then cDNA libraries were constructed. These libraries were sequenced using the Illumina platform at the Novogene Bioinformatics Technology Co., Ltd. (Beijing, China). To ensure data quality, all RNA sequence data were filtered using Fastp with default parameters [51]. Subsequently, the reads were aligned with the cultivated sandalwood reference genome using the HISAT2 version 2.1.0 software with default parameters [52]. To assess the expression levels of individual genes, the fragments per kilobase per million mapped reads (FPKM) of each gene was quantified by FeatureCounts [53]. Subsequently, differentially expressed genes (DEGs) were identified using the DESeq2 package in R, with a stringent false discovery rate (FDR) threshold of ≤0.05 and a |log2 FC| ≥ 1 criterion to establish statistically significant differences in gene expression [54].

### 2.5. Real-Time Quantitative PCR Analysis

Total RNA was extracted using the TRIzol reagent, with subsequent removal of trace amounts of genomic DNA using the RNase-free DNase I method (RNase-free; TakaRa). According to the manufacturer’s protocol, the first-strand cDNA was synthesized using a PrimerScript RT MasterMix (Takara, Kusatsu, Japan). The Primer3 web (version 4.1.1) software was used to design RT-qPCR gene-specific primers (Supplementary Table S4), and the primer sequences were synthesized by Shanghai Biosune Platinum Bio-Technology Co. (Shanghai, China) Primers SaSAUR27-1 and SaSAUR28-4 were used in the final data analysis. The data of relative gene expression were analyzed using the 2^−ΔΔCT^ method, with the actin gene (GI:156972025) of *S. album* as the internal reference to account for differences. There were three biological and three technical replicates performed for each sample. We used the GraphPad Prism version 8 software for statistical analysis and data visualization.

## 3. Results

### 3.1. Identification and Analysis of Physicochemical Properties of the SAUR Genes

The conserved domains in the SAUR proteins of *S. album* were validated using the SMART and CDD databases, resulting in the identification of 43 *SAUR* genes (Supplementary File S1). The physical locations of the *SAUR* genes on the chromosomes were determined by querying the genome annotation files and were named *SaSAUR01* to *SaSAUR43* sequentially (Table 1). The physicochemical characteristics of the 43 identified SAUR proteins were examined using the ExPASy program. The study revealed that the length of these proteins ranged from 75 (*SaSAUR15*) to 434 (*SaSAUR01*) amino acids, and their molecular weight ranged from 8.32 to 48.35 kDa. The average isoelectric point (pI) of all the SAUR proteins was calculated to be 8.37, and ranged from 4.45 (*SaSAUR28*) to 11.16 (*SaSAUR08*) (Table 1). Notably, one-quarter of the SAUR proteins were classified as acidic because their isoelectric point (pI) values were below 7, while the remaining three-quarters were alkaline (Table 1). The subcellular localization analysis of *SAUR* genes using Plant-mPLoc revealed that most *SAUR* genes were predicted to be located in chloroplasts. The remaining *SAUR* genes were predicted to be distributed in the nucleus, while only the *SaSAUR36* gene was predicted to be located in the cytoplasm (Table 1). A thorough examination of the physicochemical characteristics and subcellular localization of the identified SAUR proteins was conducted in detail to gain deeper insight into their functions in *S. album*.

### 3.2. Gene Structure and Phylogenetic Analysis of SaSAUR Genes

**Figure 1:**
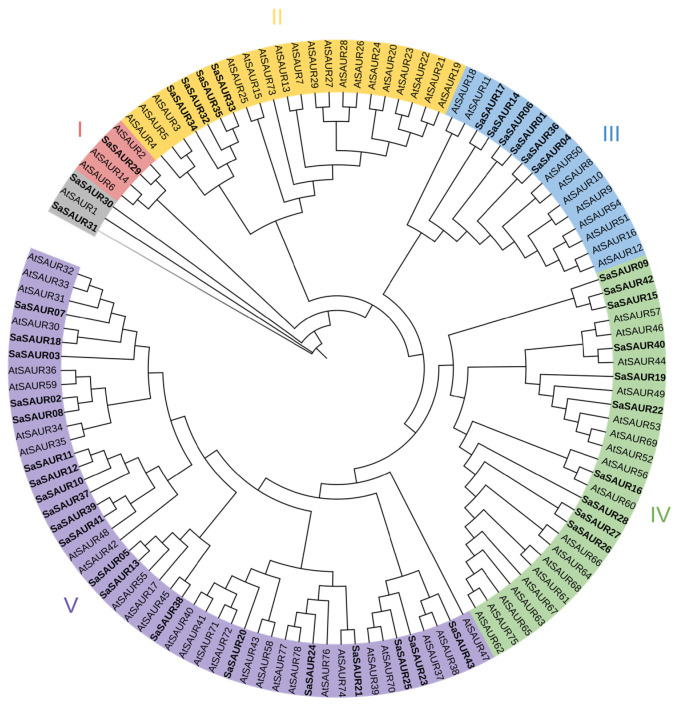
Phylogenetic tree of SAUR proteins sequences in *Santalum album* and *Arabidopsis thaliana* (different-colored shaded areas are used to differentiate distinct groups, and bold text indicates SAUR proteins of *Santalum album*).

**Figure 2:**
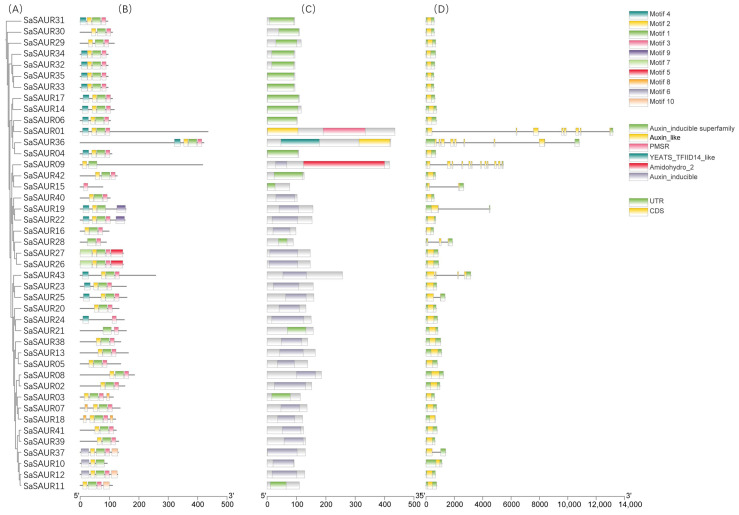
Conserved motifs, conserved domains, and gene structures of the *SaSAUR* genes. (**A**) Phylogenetic tree of the SaSAUR proteins. (**B**) Conserved motifs in the *SaSAUR* genes. (**C**) Conserved domains in the *SaSAUR* genes. (**D**) Exon–intron structure of the *SaSAUR* genes.

A phylogenetic tree was constructed for the SAUR proteins from *Santalum album* and *Arabidopsis thaliana* using MAFFT version 7 and IQ-TREE version 2 software. The results of the phylogenetic tree revealed that sandalwood *SAUR* genes could be classified into five groups. The study found that there was a total of 43 SAUR proteins, with one in Group I, four in Group II, five in Group III, ten in Group IV, and twenty-one in Group V. Additionally, there were two *SAUR* genes that could not be classified into any of the groups (Figure 1). Group V had the highest number of family members, while Group I had the lowest. In all groups, the number of *SAUR* genes in *A. thaliana* exceeded that in *S. album*. However, it is worth noting that the number of *SAUR* genes in Group V approximates that in *A. thaliana*, with 28 in *A. thaliana* and 20 in *S. album*. The conserved motifs, conserved domains, and exon–intron structure were analyzed to explore the structural diversity of *SaSAURs* (Figure 2). The online MEME tool identified 10 conserved motifs (motif 1 to motif 10) in SAUR proteins (Figure 2B). Motifs 1, 2, and 3 were widely distributed among the *SAUR* gene family in the order of 2–1–3, indicating strong conservation of the conserved domains of the *SAUR* gene family in *S. album*. Only *SaSAUR15*, *SaSAUR21*, *SaSAUR24*, and *SaSAUR28* do not contain motif 2. *SaSAUR15* and *SaSAUR24* do not have motif 1, while *SaSAUR09*, *SaSAUR10*, and *SaSAUR19* do not have motif 3. However, some motifs are unique to specific *SAUR* genes. For instance, motif 5 and motif 7 were exclusively found in *SaSAUR26* and *SaSAUR27*; motif 9 was identified in *SaSAUR19* and *SaSAUR22*; motif 6 was observed in *SaSAUR10*, *SaSAUR12*, and *SaSAUR37*; and motif 8 was specifically present in *SaSAUR03*, *SaSAUR07*, and *SaSAUR08*. Members of the same group, such as *SaSAUR32* and *SaSAUR34*, *SaSAUR04* and *SaSAUR06*, and *SaSAUR26* and *SaSAUR27*, exhibited similar motif patterns (Figure 2B). This indicates that they likely share similar functions. All *SaSAUR* genes contain conserved auxin domains, as shown in Figure 2C. In Figure 2D, the exon–intron structure of the *SaSAURs* clearly verify the diversity of the gene structures. The 43 *SaSAUR* genes exhibited different numbers of exons, ranging from 1 to 12. *SaSAUR09* has 12 exon structures, while *SaSAUR01* and *SaSAUR36* have 8 exons. Most of the *SaSAUR* genes did not contain introns. Nine (20.93%) of the *SaSAUR* genes contained one or more introns, while the remaining 34 (79.07%) *SaSAUR* genes were intron-free (Figure 2D). The similar conserved motifs and gene structures of *SAUR* genes within the same group strongly supported the accuracy of the group classification.

### 3.3. SAUR Gene Duplication and Chromosomal Location

**Figure 3:**
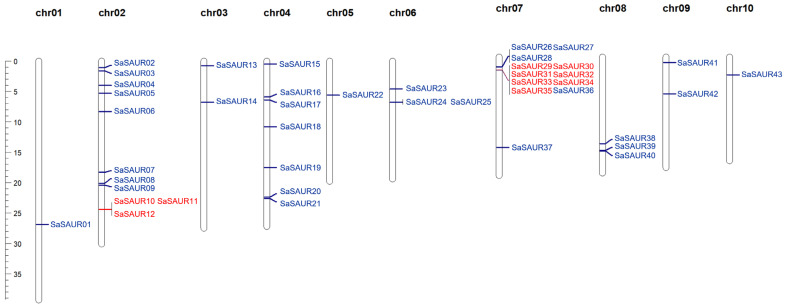
Chromosomal locations of the 43 *SAUR* genes in *S. album*. The ruler on the left indicates the physical position of the reference genome. The pair of tandemly duplicated genes is shown in red font.

The locations of the *SAUR* genes were extracted from the genome-annotated generic-feature format (GFF) file of *Santalum album*, and the physical locations of these *SAUR* genes on the chromosomes were visualized using the MapChart version 5.4.6 software. Figure 3 illustrates the irregular distribution of the 43 *SAUR* genes across 10 chromosomes. Specifically, one gene was located on chr01 (2.32% of the total), eleven genes on chr02 (25.58%), two genes on chr03 (4.65%), seven genes on chr04 (16.28%), one gene on chr05 (2.32%), three genes on chr06 (6.98%), twelve genes on chr07 (27.91%), three genes on chr08 (6.98%), two genes on chr09 (4.65%), and one gene on chr10 (2.32%). Some of the *SaSAUR* genes can be observed in Figure 3 as gene clusters.

**Figure 4:**
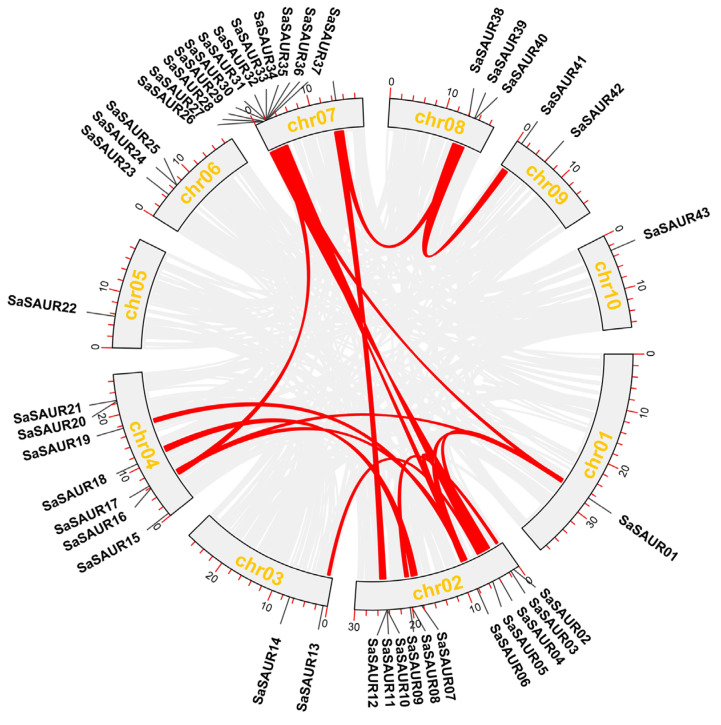
The intrachromosomal segmental duplication map in *S. album*. Gray lines are collinear blocks in *S. album*. Red lines are the syntenic gene pairs of *SaSAUR* genes.

Gene duplication events typically include tandem and segmental duplications, and they are important for studying the expansion of gene families. The results revealed the presence of 14 pairs of segmentally duplicated (SD) genes and 9 pairs of tandemly duplicated (TD) genes within the *SaSAUR* genes (Table 2). *SaSAUR* genes involved in tandem duplications typically belong to the same group. The presence of multiple duplication events in *SaSAUR* genes suggests that certain genes have arisen through both tandem and segmental duplication, potentially serving as drivers of gene evolution. The majority of segmental duplications in the *SAUR* gene family of *Santalum album* were located on chr02, chr04, and chr07, as shown in Figure 4. Ka/Ks ratios can predict the selection pressure on genes encoding proteins. Our analysis revealed that the Ka/Ks ratios of all the duplicated gene pairs were below 1, with the exception of three gene pairs where the Ka/Ks value was incalculable (Table 2). A Ka/Ks value below 1 indicates a purifying (negative) selection, while a Ka/Ks value of 1 indicates neutral evolution, and Ka/Ks values above 1 indicate adaptive (positive) selection. Our findings suggest that negative selection may have played a role in the development of these duplicated genes in the *SAUR* gene family in *Santalum album*.

### 3.4. Expression Analysis of SaSAUR Genes

**Figure 5:**
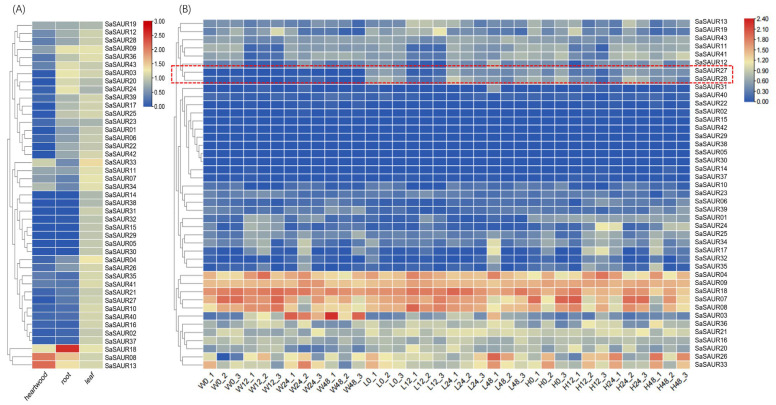
Expression profiles of the *SaSAUR* genes. (**A**) Expression of *SaSAUR* genes in various tissues (heartwood, root, leaf). (**B**) Expression of *SaSAUR* genes under salt stress. Differential genes are labeled in red boxes. The initials of the horizontal coordinates represent the salt solutions (W: CK; L: 50 mmol/L; H: 100 mmol/L), followed by treatment times 0 (0 h), 12 (12 h), 24 (24 h), and 48 (48 h), with the numbers after the underscore indicating the biological replicate. Expression profiles were normalized to log_10_(FPKM).

The current research work investigated the expression patterns of all 43 *SaSAUR* genes using transcriptome data from various tissues of *Santalum album* to understand their potential functions (Supplementary Table S2). As shown in Figure 5A, *SaSAUR* genes exhibited distinct tissue-specific expression patterns. Notably, all the *SaSAUR* genes were expressed in leaves, displaying no significant differences in their expression levels in leaves, which was consistent with the results of the subcellular localization indicating that the majority of *SaSAUR* genes are localized in chloroplasts. While approximately half of the *SaSAUR* genes did not show expression in roots, it is worth noting that *SaSAUR18* exhibited high expression levels in roots, hinting a pivotal role in the function of root development. Only a limited number of *SaSAUR* genes (*SaSAUR33*, *SaSAUR07*, *SaSAUR08*, *SaSAUR09*, *SaSAUR11*, *SaSAUR13*, *SaSAUR18*, *SaSAUR19*, and *SaSAUR34*) were expressed in the heartwood, with *SaSAUR08* and *SaSAUR13* showing significantly higher expression levels. *SaSAUR08*, *SaSAUR11*, *SaSAUR13*, and *SaSAUR18* were expressed in all three tissues, indicating their significant roles in plant growth and development.

**Figure 6:**
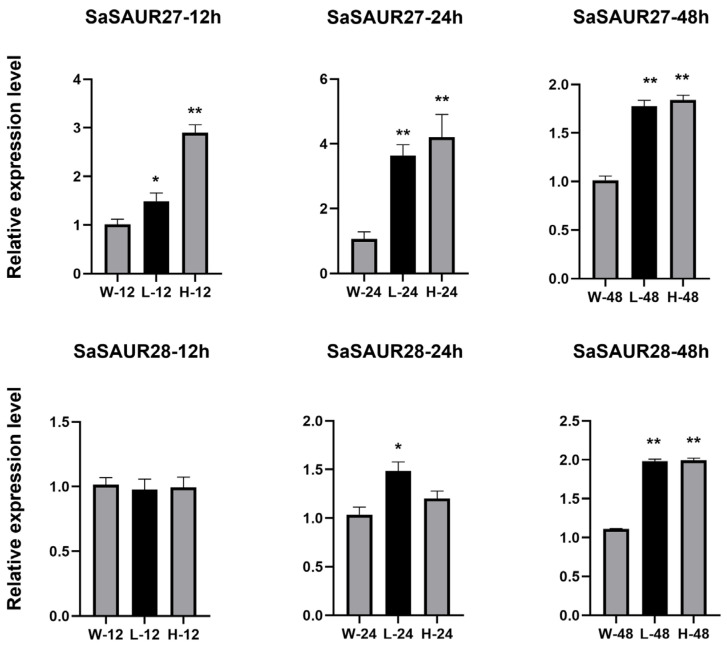
Expression profiles of *SaSAUR27* and *SaSAUR28* in response to salt treatment as determined by RT-qPCR. The *x*-axis indicates the different concentrations of NaCl solution treatments (W: CK; L: 50 mmol/L; H: 100 mmol/L), and the *y*-axis indicates the relative expression levels. The values are the means ± SD of three independent experiments, and the asterisks indicate a significant difference (* *p* < 0.05; ** *p* < 0.01) compared with the corresponding controls.

The *SAUR* gene family plays a crucial role in various abiotic stresses. To comprehend the function of the *SAUR* gene family in salt stress, we exposed seedling leaves of *Santalum album* to different salt concentrations (W: CK; L: 50 mmol/L NaCl; H: 100 mmol/L NaCl) for 0, 12, 24, and 48 h, with three biological replicates. Quality control was conducted on the data obtained from sequencing the libraries on the platform, revealing that all samples adhered to the established standards. The sequencing produced a total of 413.17 Gb of raw data, which was subsequently filtered to produce 402.13 Gb of high-quality data. The specific sequencing quality for each sample is comprehensively presented in Supplementary Table S1. An analysis of the transcriptomic data for *SaSAURs* under salt stress using a bioinformatics approach revealed that most *SaSAUR* genes were expressed both before and after salt stress (Figure 5B and Supplementary Table S3). It is worth noting that *SaSAUR04*, *SaSAUR07*, *SaSAUR08*, *SaSAUR09*, and *SaSAUR18* exhibited significantly higher expression levels. *SaSAUR27* and *SaSAUR28* are salt-stress-associated differential genes (log_2_FC > 1), with almost no expression before salt stress and significantly up-regulated expression after salt stress, indicating that these genes may play a role in the response to salt stress. The two predicted differential genes (*SaSAUR27* and *SaSAUR28*) were selected to detect their expression characteristics under salt treatment using real-time quantitative polymerase chain reaction (RT-qPCR). Figure 6 shows that *SaSAUR27* exhibited significant differences from the control at all treatment time periods after treatment with the salt solution. *SaSAUR28* did not exhibited any significant differences at the 12 h treatment time but exhibited significant differences at the 24 h and 48 h treatment times. The RT-qPCR results were consistent with the transcriptome results.

## 4. Discussion

The *SAUR* gene family has been identified in a variety of plants and shown to play a critical role in plant growth and stress tolerance [24]. Spartz found that overexpression of *AtSAUR19* in *Arabidopsis thaliana* resulted in increased expression in the leaf area, which positively regulated leaf growth by promoting leaf cell enlargement [55]. Overexpressing both *AtSAUR41* [27] and *AtSAUR63* [26] resulted in enlarged petals and elongated filaments, highlighting their significance in plant growth. In Belladonna, *AbSAUR1* promotes the growth and development of the plant, resulting in increased plant height and root meristem, which significantly enhances yield and alkaloid content [56]. The auxin-induced SAUR protein can regulate PM H^+^-ATPase activity by modulating PP2C-D phosphatase, causing an increase in plasma membrane potential, providing expansion pressure for cell growth [24]. Consequently, it promotes cell expansion, plant growth and development. The results of several studies have shown that *TaSAUR75* plays a positive regulatory role in plants’ responses to drought and salt stress [57]. In cucumber, *CsaSAUR1*, *CsaSAUR13*, *CsaSAUR15*, *CsaSAUR49*, and *CsaSAUR50* were up-regulated by high-temperature-induced expression, suggesting that they may be involved in high-temperature tolerance response processes [58]. The *OsSAUR51* gene in rice could inhibit the spread of pathogenic bacteria by reducing the accumulation of auxin, which made the plant resistant to bacterial leaf blight (BLB) and played a positive role in biotic-stress tolerance in rice [59]. Guo found that overexpression of the *TaSAUR78* gene could enhance the tolerance of wheat to salt and drought stresses [60]. Analysis of the *S. album* transcriptome data revealed that the majority of *SaSAUR* genes showed no significant changes in their expression level before and after salt stress. Expression patterns were categorized into two distinct cases: one where genes remained unexpressed both before and after salt stress, and another where genes exhibited consistent expression levels before and after salt stress. The *SaSAUR27* and *SaSAUR28* (4.65% of total) genes in *S. album* exhibited significant responses to salt stress. These two genes were classified within Group V alongside *AtSAUR36* and *AtSAUR41*, suggesting their potential functional similarities. These results are confirmed by comparative analysis with other species. Pineapple possesses 52 *SAUR* genes, with 3 genes (5.77%) exhibiting responsiveness to salt stress [18]. In contrast, alfalfa possesses 433 *SAUR* genes, of which 11 genes (2.54%) respond to salt stress [28].

Based on the genomic data of *S. album*, a total of 43 *SaSAUR* genes were identified in this study. The number of *SAUR* gene family members was significantly lower than that of peanut (162) [61], poplar (105) [35], and maize (79) [22], but higher than that of oil palm (40) [62]. The limited number of members might be associated with the extended growth cycle characteristic of *S. album*. *SaSAUR* genes were classified into five groups depending on their phylogenetic relationships, and *SAUR* genes of melons [63] have also been classified into five groups. In rice [16], tomato [22], and watermelon [64], five, three, and two clusters of *SAUR* gene family members have been identified, respectively. Chromosomal localization revealed that the *SaSAUR* genes were unevenly distributed among chr01 to chr10, with *SaSAUR26* to *SaSAUR36* clustered in the short arm of chr07. In addition, there was a high degree of sequence similarity among the members of these gene clusters. Four genes (*SaSAUR32*, *SaSAUR33*, *SaSAUR34*, *SaSAUR35*) on chr07 were classified in group II, and these genes were clustered together on the same branch of the phylogenetic tree, exhibiting a high level of similarity in motif arrangement and gene structure. In terms of gene structure, the *SAUR* genes contain almost no introns, but AUX and GH3, which also belong to the three large early auxin-responsive families, have a high number of introns. The number and location of introns can often influence the level of gene expression. The incidence of alternative splicing in intron-less genes tends to be low, so the *SAUR* genes may be relatively functionally conserved. The identified members of *SAUR* gene family with intronic structures accounted for 20.9% of the total, which is higher than maize (7.5%) and poplar (10.5%), and similar to that in millet (18.1%). Segmental and tandem duplications are the primary drivers of gene family expansion and evolution [65]. We identified nine tandem duplications and 14 segmental duplication events in 43 *SaSAUR* genes (Table 2). This suggests that segmental duplication plays a critical role in amplifying and evolving the *SAUR* gene family of *S. album*. Motif 1, Motif 2, and Motif 3 were widely distributed among the *SaSAUR* genes and were arranged in the order of 2–1–3, which is consistent with the study of the cabbage *SAUR* gene family by Zhao [66]. The exon–intron structures and motif arrangements were highly conserved, and the genes of close relatives exhibited similar genetic structures. The similarity and variation in gene structure, motifs, and conserved domains of *SaSAUR* genes are certainly related to the evolutionary and gene duplication of *S. album*. Previous studies documented the subcellular location of *SAUR* genes within the nucleus [67] and cytoplasm [68]. Contrary to these findings, our study indicated that a predominant fraction, exceeding 70%, of the SaSAUR proteins was predicted to be localized within the chloroplasts, a pattern similarly observed in maize [69]. Plant chloroplasts are important intracellular organelles that produce ATP (Adenosine triphosphate) through photosynthetic phosphorylation, suggesting that auxins may play a role in the energy transfer process that takes place at the chloroplast membrane [70].

To explore the principal functions of *SaSAUR* genes and the role of salt stress on them, we embarked on an expression analysis across distinct tissues—roots, leaves, heartwood—and at incremental salt exposure durations (0, 12, 24, and 48 h), leveraging transcriptome datasets. A meticulous tissue-specific expression scrutiny unveiled that disparate *SAUR* genes assume unique roles in the ambit of plant growth and morphogenesis. Notably, the expression profile of *SAUR* genes in the leaves of *S. album* was significantly higher than in other parts of the plant, a pattern similar to that observed in watermelon [64]. This suggests a conserved role of *SAUR* genes in foliar development across species. Specifically, *SaSAUR27* and *SaSAUR28*, both localized on chr07 and categorized under group IV, emerged as salt-stress-responsive genes (log_2_FC > 1), suggesting their potential involvement in the salt-stress response mechanism. Subsequent RT-qPCR analysis results were consistent with those of the transcriptome. *SaSAUR27* differed from the blank control group at every time interval, and *SaSAUR28* also showed differential performance at all time intervals, except at 12 h. This pattern intimates a robust involvement of these genes in salt-stress adaptation. This study has revealed the expression response of *SaSAUR* genes in different tissues and under various salt treatments. With the advent of comprehensive plant genomic sequencing, it is anticipated that an expanded repertoire of *SAUR* genes will be identified across diverse taxa. These findings will contribute to a more in-depth study of the functional characterization and the evolutionary genesis and diversification of the *SAUR* gene family.

## 5. Conclusions

The *SAUR* gene family in *Santalum album* was comprehensively analyzed for the first time in this study. A total of 43 *SaSAUR* genes were identified, which were unevenly distributed across 10 chromosomes. The phylogenetic tree and gene structure analyses revealed that *SaSAUR* genes can be classified into five groups, each exhibiting similar gene structures. Additionally, most *SaSAURs* were found to lack introns. The duplication events of *SaSAUR* genes suggests that segmental duplication served as the primary means of evolutionary expansion for *SAUR* gene family. Analysis of *SaSAUR* gene expression levels in various tissues and under salt-stress conditions uncovered that all *SaSAUR* genes are expressed in leaves. *SaSAUR08* and *SaSAUR13* exhibit significantly higher expression levels in heartwood compared with roots and leaves. Conversely, *SaSAUR18* shows significantly higher expression in roots than in heartwood and leaves. Additionally, *SaSAUR27* and *SaSAUR28* have emerged as potential differential genes in response to salt stress. Overall, our study will contribute to a better understanding of the complexity of the *S. album SAUR* gene family and will serve as a valuable guide for future functional analysis studies.

## Figures and Tables

**Table 1:** Detailed information on the *SAUR* gene family in *S. album.*

Gene Name	Gene Id	Length AA	Molecular Weight (kD)	pI	Chr	Predicted Localization
*SaSAUR01*	SA01G02339	434	48.35	8.96	1	Chloroplast
*SaSAUR02*	SA02G00132	151	17.13	10.66	2	Chloroplast
*SaSAUR03*	SA02G00206	112	12.59	6.33	2	Nucleus
*SaSAUR04*	SA02G00516	107	11.81	9.34	2	Chloroplast, Nucleus
*SaSAUR05*	SA02G00677	137	15.15	9.40	2	Chloroplast
*SaSAUR06*	SA02G01106	102	11.18	8.56	2	Chloroplast
*SaSAUR07*	SA02G02406	135	15.60	8.45	2	Nucleus
*SaSAUR08*	SA02G02629	184	20.35	11.16	2	Chloroplast, Mitochondrion
*SaSAUR09*	SA02G02667	416	46.60	8.76	2	Chloroplast
*SaSAUR10*	SA02G03132	91	9.90	9.67	2	Chloroplast
*SaSAUR11*	SA02G03133	109	12.41	4.99	2	Nucleus
*SaSAUR12*	SA02G03134	127	13.95	9.07	2	Chloroplast
*SaSAUR13*	SA03G00088	163	18.15	9.48	3	Nucleus
*SaSAUR14*	SA03G00867	115	13.31	10.02	3	Chloroplast
*SaSAUR15*	SA04G00058	75	8.32	10.28	4	Chloroplast, Nucleus
*SaSAUR16*	SA04G00729	97	11.43	9.58	4	Chloroplast, Nucleus
*SaSAUR17*	SA04G00792	109	12.09	9.75	4	Chloroplast, Nucleus
*SaSAUR18*	SA04G01353	120	14.15	6.34	4	Nucleus
*SaSAUR19*	SA04G02219	155	17.14	9.72	4	Chloroplast, Nucleus
*SaSAUR20*	SA04G02816	131	14.82	8.71	4	Chloroplast
*SaSAUR21*	SA04G02839	156	17.35	8.58	4	Chloroplast, Cytoplasm, Nucleus
*SaSAUR22*	SA05G00696	152	17.17	8.82	5	Chloroplast, Nucleus
*SaSAUR23*	SA06G00626	156	17.87	9.85	6	Chloroplast
*SaSAUR24*	SA06G00887	149	15.81	6.50	6	Nucleus
*SaSAUR25*	SA06G00889	158	17.41	5.38	6	Nucleus
*SaSAUR26*	SA07G00208	146	16.17	8.72	7	Nucleus
*SaSAUR27*	SA07G00209	146	16.26	8.33	7	Chloroplast, Mitochondrion, Nucleus
*SaSAUR28*	SA07G00210	88	9.63	4.45	7	Nucleus
*SaSAUR29*	SA07G00277	115	12.73	8.01	7	Chloroplast, Mitochondrion, Nucleus
*SaSAUR30*	SA07G00278	109	12.58	6.29	7	Chloroplast, Nucleus
*SaSAUR31*	SA07G00279	93	10.20	6.82	7	Chloroplast
*SaSAUR32*	SA07G00280	94	10.76	8.96	7	Chloroplast, Mitochondrion, Nucleus
*SaSAUR33*	SA07G00281	94	10.73	8.93	7	Chloroplast
*SaSAUR34*	SA07G00282	94	10.59	6.57	7	Chloroplast
*SaSAUR35*	SA07G00283	94	10.74	9.37	7	Chloroplast, Nucleus
*SaSAUR36*	SA07G00284	420	47.02	7.71	7	Cytoplasm
*SaSAUR37*	SA08G01800	137	14.98	9.10	8	Chloroplast, Cytoplasm, Nucleus
*SaSAUR38*	SA08G01915	130	14.92	6.33	8	Chloroplast
*SaSAUR39*	SA08G01928	101	11.61	8.31	8	Nucleus
*SaSAUR40*	SA09G00119	123	14.21	9.26	9	Chloroplast
*SaSAUR41*	SA09G00719	119	13.98	5.61	9	Chloroplast
*SaSAUR42*	SA09G00803	125	14.33	9.18	9	Nucleus
*SaSAUR43*	SA10G00348	256	28.87	9.66	10	Chloroplast, Nucleus

**Table 2:** Ka, Ks, and Ka/Ks of duplicated gene pairs of the *SaSAURs.*

Duplicated Gene Pairs	Non-Synonymous (Ka)	Synonymous (Ks)	Ka/Ks	Duplicated Type
SaSAUR10&SaSAUR11	0.20	0.45	0.44	tandem
SaSAUR11&SaSAUR12	0.22	0.48	0.46	tandem
SaSAUR26&SaSAUR27	0.10	0.33	0.32	tandem
SaSAUR29&SaSAUR30	0.42	NaN	NaN	tandem
SaSAUR30&SaSAUR31	0.35	NaN	NaN	tandem
SaSAUR31&SaSAUR32	0.30	1.46	0.21	tandem
SaSAUR32&SaSAUR33	0.12	0.94	0.13	tandem
SaSAUR33&SaSAUR34	0.10	0.33	0.30	tandem
SaSAUR34&SaSAUR35	0.11	0.31	0.35	tandem
SaSAUR01&SaSAUR04	0.22	1.88	0.12	segmental
SaSAUR01&SaSAUR06	0.14	0.80	0.17	segmental
SaSAUR01&SaSAUR17	0.30	2.11	0.14	segmental
SaSAUR01&SaSAUR36	0.20	NaN	NaN	segmental
SaSAUR04&SaSAUR06	0.21	1.81	0.11	segmental
SaSAUR02&SaSAUR08	0.25	0.51	0.48	segmental
SaSAUR05&SaSAUR13	0.41	1.71	0.24	segmental
SaSAUR07&SaSAUR18	0.32	1.54	0.21	segmental
SaSAUR06&SaSAUR17	0.26	1.61	0.16	segmental
SaSAUR06&SaSAUR19	0.62	1.59	0.39	segmental
SaSAUR04&SaSAUR36	0.12	1.60	0.07	segmental
SaSAUR06&SaSAUR36	0.18	1.43	0.12	segmental
SaSAUR16&SaSAUR26	0.56	3.45	0.16	segmental
SaSAUR38&SaSAUR40	0.23	1.00	0.23	segmental

## Data Availability

The original contributions presented in this study are included in the article and Supplementary Material. The whole genome sequence and annotation files of *S. album* can be downloaded from the National Genomics Data Center of China at https://ngdc.cncb.ac.cn/gwh/Assembly/37800/show (accessed on 3 April 2024). The RNA-seq data under salt stress of *S. album* can be downloaded from the National Genomics Data Center of China at https://bigd.big.ac.cn/gsa/browse/CRA015677 (accessed on 3 April 2024).

## Supplementary Materials

The following supporting information can be downloaded at: https://www.mdpi.com/article/10.3390/plants13101286/s1.
