# Supplementary material for: Genome-Wide Analysis of the SAUR Gene Family and Its Expression Profiles in Response to Salt Stress in Santalum album
Source: Plants (Basel). 2024 May 7;13(10):1286. doi: 10.3390/plants13101286 (PMC11125248; doi:10.3390/plants13101286)
Supplement: Supplementary file 1 [file plants-13-01286-s001.zip › Supplementary File S1.pdf]

Supplementary S1. The protein sequences of 43 SAUR genes in *Santalum album*

>SA01G02339.t1 gene=SA01G02339 SaSAUR01
MAIRKPNRVCQTECLKQILKRCSSFGKKHGYASIEDGPPGDVPKGHFAVYVGENRSRYIV
PISWLAHPEFQSLLRRAEEEFGFDHGMGITIPCEEVAFLSLATMIAAGNRTRVCTVAGYYS
TTRPLASPRLCVPKSQTEGENPMNNDALFVPLILSFILLIYFMAGRALCIRIPDRMPEPSKD
RSIHPSQTAVFALGSFWRSEAAFGCLDGVVRTTSGYAGGSKINPQYRSLGEHAESVQIEY
DPGVISFKQLLEVFWSSHDPTQVFGPGPDVSNQYRSIIFTNGTEESRLAAVSKERQQTKSR
KSILTTQIQQLEMFYSAEPEHQKFELKRSPFMLQLIGNLPQEELEKSNLAAKLNGYAAELC
PAKIQMQIDTKISDIIGKGWPILREIFDFALFKIGFCSSPAGKLAILKRAQAGRAPKPYEAFK
ALKLIP
>SA02G00132.t1 gene=SA02G00132 SaSAUR02
MRTLNGFKLMKRVRTLYRKRTRHFSRIHSWGHRLKSALRSGRSFLQDSVSGSSRRYVSIG
AAAVEEKPKQTVPKGHLAVYVGGDLKRVLVPVMYFNHPLFGELLREAEKEYGFYHPGG
TIPCRVSEFERVQTSIAAVHGGQHFAGSRCLL
>SA02G00206.t1 gene=SA02G00206 SaSAUR03
MGYGESGVESNKLRGVPKGCLAITVGGTGEEPERLVVPVSYLNHPRFVELLKEVEEEYGF
DHHQGTITIPCPAQDFLSLRTMIHDEHSSSSLNHHHHRHHRHLRHLVGCFGV
>SA02G00516.t1 gene=SA02G00516 SaSAUR04
MALRKSSKALPQAALLKQILKRCSSLKKRHGSGGGDDLPLDIPKGHFAVYVGENRIRHIV
PISLLAHPQFQGLLHRAEEEFGFDHEMGLTIPCKEDLFRSLTSTLAG
>SA02G00677.t1 gene=SA02G00677 SaSAUR05
MDSVKKRWKKLSIITRALQGFRSMAPKAKPPPPAGCFVVYVGEEKERFFVKTKWASHPF
FQILLEDAESEYGYSSQGPILLPCNVHLFRRVLAAMEVSDGDEADALAAGAAFGGGLLRS
ARSYTPLRCSPALKLNQF
>SA02G01106.t1 gene=SA02G01106 SaSAUR06
MAIRKFNRVPQTAALKQILKRCSSFGKGGGADHCPSDVPKGHFAVYVGENRARYIVPISW
LAHPGFQMLLQRAEEEFGFGHGMGITIPCEEDAFQSLASMIG
>SA02G02406.t1 gene=SA02G02406 SaSAUR07
MGSGERSFRSFHLHLPHLHHPHHHHHQGHGGHYGKRQGGNRDRIRDVPKGCLAIRVGG
KGEEPQRFVVPVAYFNHPLFLQLLKEAEEEYGFDHKGTITIPCQVRDFRFVQCIIDEERSSS
SMHLHHHDVIGCFRV
>SA02G02629.t1 gene=SA02G02629 SaSAUR08
MTKLGGFKLARRIKTLCRRIIRRRIPTFRAGFHRLDPPDCRARSSSFSSTVSKVCSWGHRLK
TRAKSAICSASRPGVGLLPGPGLGAGRGYVPIGGEKKPGAVPKGHLAVYVGQNDGDFQR
VLVPVMYFNHPLFGQLLREAEEVYGFQHPGGITIPCRISEFESVQTRIAAVHGYRRILRRQ
AL
>SA02G02667.t1 gene=SA02G02667 SaSAUR09

MEERKKKMKVKKGWLAVQVGLDEDGGGSRRFMIPIGYLYHPLFQRLLERSREIYGYDTA
GPLRLPCSRLNPLISTLVASRRLKPPRPSVSSSPKQSRFRIIAAKMATIQDDDNKIAPSPIKIF
DSHLHVWASPQEAADKYPYFPGQEPTLPGNVDFLLQCMEEAGVDGALVVQPINHKFDHS
LVTSVLRNYPSKFVGCCLANPAEDGSGVQQLEHLVTKDGYRAVRFNPYLWPSGQQMTN
EIGKAMFSKAGELRVPVGFMCMKGLNLHISEIEKLCSEFPSTVVLLDHLAFCKQPTNEEEN
SALSKLLKLSRFPQVYVKFSALFRVSKMPFPYKDLAPLLFQVVSTFGANRVMWGSDFPFA
VPECGYREAKEAVSIIADQVPLSSSELEWIMGRTALQLFQGLSYPSLPSALSNEF
>SA02G03132.t1 gene=SA02G03132 SaSAUR10
MAKKMGGGIVKLKTVIEKLQRSFSGEKKAANSAVPKDVKDGHFVVMAANDNEHRRFIV
PLSYLVQPAFLGLLKDAAEVYGINQKGVLTIPC
>SA02G03133.t1 gene=SA02G03133 SaSAUR11
MAAYYIVSKDVKEGHLAVTAAEDDEQRRFIVPLSYLVKPVFLGFLEDVAEVYSFNQKGK
MMIPCRPCELERLLAEQSRIDKDSNDVAVKLGFIQSFDQKLRMSMEEGTA
>SA02G03134.t1 gene=SA02G03134 SaSAUR12
MAKRMGGGIVKIKVVIAKLQRSLSGKKMAANFVVPEDVKEGHFAVIAAIDDEQRRFIVPL
SYLAQPAFLGLLEDAANVYGFDQKGALMIPCRPCELERLMAEQSRGDRDSSDVAVKWG
SYKASIRSC
>SA03G00088.t1 gene=SA03G00088 SaSAUR13
MDGGIKEKGKRGLFNKTWERCRSIGRGGAVKRASSSPPPPPLRLGAKSKSWPRLPGKQQ
KRVAPEGCFSVYVGPQKQKFVVKTEYANHPLFRMLLEEAELEYGYNSQGPLLLPCDVDL
FYRVLMEMDCDDGDEGIGRRGGGGCAFGSPYRLLNPSRSLLMNHF
>SA03G00867.t1 gene=SA03G00867 SaSAUR14
MAIIRKAKTETSSLKQILKRCSSALWGRRRRRNRNEYGPPEDVPEGHFAVYVGENRKRHV
VPITILRRPEFQRLLRRAEEEFGFDHCMGGLTIPCEDPFFRTLTSAPLTVKSGEP
>SA04G00058.t1 gene=SA04G00058 SaSAUR15
MSAPEFGYDRKGPLSIPCEAPCIRNILQRRIIVMRTLQSFTLTSVHNAIYGFSQLTPAPHLSV
MGLRINSASPPR
>SA04G00729.t1 gene=SA04G00729 SaSAUR16
MRRTSQKLSDVVEKWRKGKKGCFVVYTKEGKRFVLPLHYLDHPIFRVLLEMAEDEFGST
VRGPLKVPCEERLMDYILCLLKRNPPKQWQTQSFGLLA
>SA04G00792.t1 gene=SA04G00792 SaSAUR17
MAIVGKASKVSAQTSPLKQILKRCSTLTKNSSSSSNNNKPGSDVPKGHFAVYVGENRARH
IVPISFLSRPEFQRLLRQAEEEFGFDHDMGLTIPCEEVAFRALKSTLMR
>SA04G01353.t1 gene=SA04G01353 SaSAUR18
MGIGQRNPLNFHLNHHHHHHHDVYDDGGDEDGEMIPKGFVGIRVGEEHRRFVVPVAYV
NHPLFMKLLNEAEAEYGFDREGPITIPCHIQEFQHVQGIIHKDNILHHHHHHHHHHFLCFR
P
>SA04G02219.t1 gene=SA04G02219 SaSAUR19

MSAGFGKCSKIRHIVRLRQLLRRWRNKAASSSRVASANRIPSDVPAGHVAVSVGSERRRF
VVPATHLNHPAFRKLLAQAAEEYGFGNNAGPLAIPSCDESVFEEVLRLISLSEPGNNPTQL
ANLDDLQRYCHTGIRTAWYESQPLLLELAGNSIW
>SA04G02816.t1 gene=SA04G02816 SaSAUR20
MKQLIRRFSRVADSSQYCMLRSPEEPQRRAARSAASFRAGSFRARRSAAVPGGHLPVYV
GDEMERFVVSAELLNHPIFVNLLNKSAQEYGYEHKGAIRIPCHVIAFERLLEALRLGSDPT
ELQDLIDDSFL
>SA04G02839.t1 gene=SA04G02839 SaSAUR21
MKKNLNSILRKCKSLSMELGRSSPYTSLRRSDSTKLLGVWGGVGRRSRRSRLADGGGDD
DGYGEEYNETVIYVGSKRRRYVVNPEFLSHPLLSALIEKSSSDERGEAAAAAAGNRRHIW
VRCEVVLFDHLLWMLQSGDPGLGSGDSLEELAELYVF
>SA05G00696.t1 gene=SA05G00696 SaSAUR22
MTSGLGSCRKIRHIVRLRQMLRRWRSKAVSARVSSPGRVPADVPAGYVAVSVGAGCKRF
VVRASHLNHPVFQKLLTQAEEEYGFTTEGPLWIPCDESVFEDVLRFLSRSDSPSSAGFVDL
EEDFQRFCHVGIMANWLESR
PLLHELTDKSIW
>SA06G00626.t1 gene=SA06G00626 SaSAUR23
MSKPSMAGAAKVDKIRQVVRLKQVMQRWRTMSLRRHRDSDSSDTTRRIPSGFLAVYVG
ADRRRFVIPTRYLNLPVFVALLDEAAEEFGFHPSGGLTLLCEIGFFEQILKLLETDEKRFGG
VELNEFSKLVSEIRRDPCNETTTDRRGFTPLLKARV
>SA06G00887.t1 gene=SA06G00887 SaSAUR24
MAKGGGGAAAGKLTKLKSVMKKWQSFSSRLGCVPNAAAGAATSSISAGADDYDGAAIS
REDTENLRPVYVGKSRRRYLVSPDVVQHPLFQELVQRPSSSPPPPSSGADAGITVACEVVL
FDHLLWMLQIADSQSHHSDSLDELVHFYAC
>SA06G00889.t1 gene=SA06G00889 SaSAUR25
MDENGGGKVTGIRQIVRLKEILQKWQSVTLSSKGSDQHPGGIAPVTNKQLKTPMCMCDS
DDETGQNPESPPDVPKGYLAVYVGPELRRFIIPTGYLSHSLFKVLLEKVEEEFGFDHSGGL
TIPCEIETFKYLLKCMENQQKAHPDEITAAGKSLSIAE
>SA07G00208.t1 gene=SA07G00208 SaSAUR26
MISLKKLIKLARKWHKVAAAKRKRISSQTINGSTDSDASTSSVASKGHFVAYSADGRRFV
ISLLYLSNTIFKELFRMSEDEFGLPSEGPITFPCESVFLEYIISFIQGPAAKDLESALLLSLANS
HHPSSRLCHEQTNEHLLLCAC
>SA07G00209.t1 gene=SA07G00209 SaSAUR27
MISLMKLFKFARKWHQVAAAKRKRISSPRTNGSMDVDNCTSSVASKDHFVLYSTGGGRF
IIPLLHINNTIFRELFRMSDDEFGLPSEGPITFPCDSVFMEYIISFIQGPAAKDLEGALQLSLA
NSHHPSSRLCHGQANEHLLLCAC
>SA07G00210.t1 gene=SA07G00210 SaSAUR28
MKISHRISSFESCRSWILNQSVRSEAVHDSLGVSQQCDFRVLFEMAEEEFGIPTDGQITLPC

NEAFLDDDGQAGVVHSSGDEGGRMAA

>SA07G00277.t1 gene=SA07G00277 SaSAUR29

MVRGIGIPTMILNAKRALKHLHRSHELGDVPKGHVAVYVVGGGEEEVRQMKRYVVPIST

LNHPTFSGLLRRAEEEFGFDHPMGGLTIPCEEHTFLQLASRLTQSSSSNMSLNIPP

>SA07G00278.t1 gene=SA07G00278 SaSAUR30

MGIGELAVEVVLHAKQAIQRRSSWRRQSRSPSNQAPTESEVPKGHFAIYVGVEEEEEKRF

VIPIAYLKHPMFQHLLGLAEDEFGFDHRMGRLTIPCSEHEFLNRISRLD

>SA07G00279.t1 gene=SA07G00279 SaSAUR31

MGIRLNAKQILQRTLSMKNGTVSGDVPKGHIAVYVGEGGKTRFVIPITYLNHPLFQNLLN

VAEEEFGFNHPMGGLTLPCSEEYFTSLTSLLSS

>SA07G00280.t1 gene=SA07G00280 SaSAUR32

MGFLLPRTCHARRILRRVLSITRTIDVPKGHFVVYVGEACRKRFVVPISYLKHPSFKDLLA

HAEEEFGFDHPTGGLTIPCKVEEFIDLTHSLSF

>SA07G00281.t1 gene=SA07G00281 SaSAUR33

MGLRLPSTFRAKQIFRRVLSDAHTIQVPKGHFVVYVGEVCKKRFVVPISYLKNPSFQDLLT

HAEKEFGFDHPMGGLTIPCRVEEFIDLTHSLSC

>SA07G00282.t1 gene=SA07G00282 SaSAUR34

MGLHLPTTFPAKQIFRRVLSSSRSISVPKGHFVVYVGEVCKKRFVVPISYLKHPSFQDLLV

HAEEEFGFDHPMGGLTVPFGEEDFIDLTHSLSC

>SA07G00283.t1 gene=SA07G00283 SaSAUR35

MGLRLPTKFPGKQIFQRALSNHRTISVPKAHFVVYVGRVCKKRFVVPISYLKDPSFQDLLA

HAEKEFGYDHPMGGHTIPCRVEEFIDLTHSLSC

>SA07G00284.t1 gene=SA07G00284 SaSAUR36

MTSGSSIEENGPDHSSFLPKITSPKMVKSAEDDEKKGLYKRLKDVEVSVPVVYGNVAFWL

GKKASEYQSHKWTVYVRGATNEDLGVVIKRVIFQLHSSFNNPTRVVESPPFELSECGWGE

FEIAITLCFQSDVSDKPLNLYHHLKLYPEDESGPLSTKKPVVVESYNEVVFSEPSEAFLARV

QSHPAVTMPRLPAGFSLPTHVPGEDANKKRGDTKDSSLSQWFTNFSEADELLKLATARQ

QLAPDYIMFCTMSTGVSSDTRPKPWYMSLRIKVFLYDHRVHVNKAGRLFLSSQTLLLFQA

SGTHKTPSQTTMAIRKSNKVLPQAAVLKQILKRCSSLGKKNGGGYGDDGLPLDVPKGHF

AVYVGENRIRHIVPISVLSHPEFQNLLHRAEEEFGFDHEMGLTIPCEETSFRSLTSMLLR

>SA08G01800.t1 gene=SA08G01800 SaSAUR37

MMMGMNKIKSFKKLAKKKVKGLAGDDNSRPSASEVLLQAADAGGEPLAKAASPLKRPR

AGFLAVYVGDERRRFVVPTGFLAHPLFRILLEKASEEFGFEQRNGIVFPCSVSTFQEVMSA

VECCNGNFDIGRLVEEIV

>SA08G01915.t1 gene=SA08G01915 SaSAUR38

MSMPTKNKEERKRALLRLKLFVGKLQRGLTLFSSPRMPDFSNADIEEELGHEERAVPDDV

KEGQFAVFAVNCGEAKRFIIELEHLSNPAFLELLEQAKEEYGFSQKGALAVPCHPEELKRI

LRDGRQKRE

>SA08G01928.t1 gene=SA08G01928 SaSAUR39
MRSKVSPSSVCDKFWQWTVWPSINEESFIPTDVPKGHLVVYVGDNCKRFVIKVTLLKHPL
FRALLDKAQDEYDFTTDTKLCIPCDERIFLSVVRCAGSRQG
>SA09G00119.t1 gene=SA09G00119 SaSAUR40
MKNREGRKRCFLWFGLFIGKLQRGLPLFSYSRLPSFSATDDMEEAMTVPLDVKKGHFAV
FAVKGGETKRFVIELEHLTNPAFLSLLELAKEEYGFSQKGVLAVPCRPEELRRILRDRMLF
SQE
>SA09G00719.t1 gene=SA09G00719 SaSAUR41
MLIRRPSMSRHGYRKLHGDGGEEAEAPVRVVVGKEKREFLVEPFVLEESPFRVLMEAAS
ARPRKEDRRGRLGEEGERRAVFVDVDSILFEHMLWLMYNDCSSLFRLNLEEIIDFYAHDF
>SA09G00803.t1 gene=SA09G00803 SaSAUR42
MNAALCSENQEKRIHSELAVASERMGKKASSMAYARKGDGARKHEHGRGDWQRVRKG
YVPVMVGIEEEEEKKERVLIPIRFINHPFMAALLEISYPELGYCHHQGPLTIPCDPHCLRRV
LRRILS
>SA10G00348.t1 gene=SA10G00348 SaSAUR43
MESKKSNKIRDIVRLQQILKKWKKLANASKSDNSNSNNNNNVGGCNKSINKFLKKTLSLS
DLGSISGTNEAPPPPKGFLAVCVGKELKRFVIPTEYLGHRAFHALLREAEEEFGFQQEGVL
KIPCEVPVFENILEMVRDKREVFFFHREDFGIATNGSGTDCYCSSDSLSERQMIPTKTDLSR
NDPQEGRQVKAVLYRQVGHQLRQWTRGSIEAFRIGARSSIVQVTTEPRSAHTAGRRPKPY
RVIVKQSNIESHA
